# The specialist in regeneration—the Axolotl—a suitable model to study bone healing?

**DOI:** 10.1038/s41536-022-00229-4

**Published:** 2022-06-30

**Authors:** A. Polikarpova, A. Ellinghaus, O. Schmidt-Bleek, L. Grosser, C. H. Bucher, G. N. Duda, E. M. Tanaka, K. Schmidt-Bleek

**Affiliations:** 1 Research Institute of Molecular Pathology, Vienna, A-1030 Austria; 2 Julius Wolff Institute and BIH Center for Regenerative Therapies, Berlin Institute of Health at Charité – Universitätsmedizin Berlin, Berlin, DE-13353 Germany

**Keywords:** Bone, Regeneration

## Abstract

While the axolotl’s ability to completely regenerate amputated limbs is well known and studied, the mechanism of axolotl bone fracture healing remains poorly understood. One reason might be the lack of a standardized fracture fixation in axolotl. We present a surgical technique to stabilize the osteotomized axolotl femur with a fixator plate and compare it to a non-stabilized osteotomy and to limb amputation. The healing outcome was evaluated 3 weeks, 3, 6 and 9 months post-surgery by microcomputer tomography, histology and immunohistochemistry. Plate-fixated femurs regained bone integrity more efficiently in comparison to the non-fixated osteotomized bone, where larger callus formed, possibly to compensate for the bone fragment misalignment. The healing of a non-critical osteotomy in axolotl was incomplete after 9 months, while amputated limbs efficiently restored bone length and structure. In axolotl amputated limbs, plate-fixated and non-fixated fractures, we observed accumulation of PCNA^+^ proliferating cells at 3 weeks post-injury similar to mouse. Additionally, as in mouse, SOX9-expressing cells appeared in the early phase of fracture healing and amputated limb regeneration in axolotl, preceding cartilage formation. This implicates endochondral ossification to be the probable mechanism of bone healing in axolotls. Altogether, the surgery with a standardized fixation technique demonstrated here allows for controlled axolotl bone healing experiments, facilitating their comparison to mammals (mice).

## Introduction

Bone fractures are one of the most common traumatic injuries, and the incidence of fractures is rising due to the ageing demographic and higher sports activity. Though most small fractures heal within weeks, 5–10% of long-bone fractures lead to delayed bone healing or non-unions (pseudoarthrosis) 6–8 months after injury^1–3^. Presently used treatments, such as intramedullary nails or fixator plates, autologous bone grafts and BMP-based therapy, do not always provide complete bone restoration^4–6^. Current studies are focusing on elucidating the cellular and molecular mechanisms of bone healing with an aim to potentiate bone regeneration in fracture non-unions.

Rodent systems, such as mouse and rat femur fractures, are commonly used in bone fracture healing studies. From studies on mammals and human, several stages of bone healing were defined. The initial stage of healing is the inflammatory response, taking place in the first days post-fracture. Immune cell signaling and clot factors attract mesenchymal progenitors to the fracture site. In the rigid-fixated fractures with small gap, direct bone formation from the mesenchymal progenitors, intramembranous ossification, takes place. In less stably-fixated fractures with a bigger gap, endochondral ossification takes place with formation of a callus, intermediate cartilaginous tissue, preceding bone formation. Soft callus consists of SOX9-expressing cartilage progenitors, later differentiating into hypertrophic cartilage. Then, due to vascularization, apoptosis of cartilage cells and invasion of osteoblasts, callus transforms into woven bone. In the remodeling phase, woven bone is gradually substituted by lamellar bone^7^.

A model of fracture non-union, critical sized defect, was developed in mammals^8,9^. The size of the non-healing fracture largely depends on the fixation method and bone size. Various techniques are being tested to improve healing of the large bone defects of critical size (critical size defect, CSD), such as artificial and natural material scaffolds, mesenchymal stromal cell transplantation and molecular factor application^6,8,9^.

One possible strategy to improve the bone regeneration is to learn from the highly regenerative organisms, such as *Ambystoma mexicanum* (axolotl). Axolotl is able to regenerate large parts of the lost appendages, including a complete patterned skeleton. In the past years, limb regeneration was studied in detail, and the cellular and molecular mechanisms were partially revealed. Induced by signaling from the wound epidermis and injured nerves, connective tissue cells of the stump migrate to the amputation plane and form a blastema, a limb bud-like mass of dedifferentiated mesenchymal progenitors^10–15^. The blastema grows and differentiates, and eventually gives rise to a scar-free, completely regenerated limb with all skeletal elements^14,16^. It was recently found that two distinct connective tissue cell types regenerate the axolotl bone after amputation. Periskeletal cells migrate along the surface of the cut bone and build the region proximal to the injury, whereas soft connective tissue (SCT) cells from the muscle interstitium and dermis migrate further to build the bone’s distal region^17,18^. Despite the ability to regenerate an amputated limb or femorotibial joint defects, axolotls cannot heal CSD^19–22^. This paradox has not been solved yet, and comparison of fracture healing and formation of bone from non-bone cells in the regenerating limbs can provide new insights in the bone regeneration processes.

**Fig. 1:**
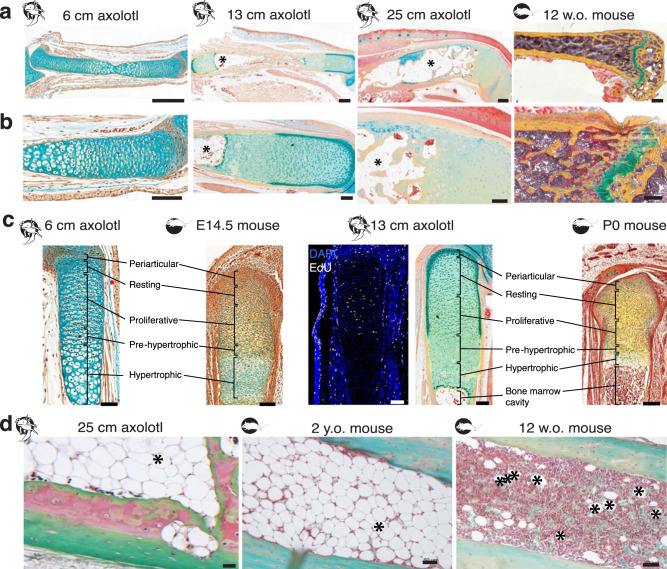
Comparison of bone structure in young versus old axolotl and axolotl versus mouse. **a** The upper hind limb samples of 6 cm, 13 cm and 25 cm snout-to-tailtip axolotl and 12 weeks-old mouse (Movat’s pentachrome staining). In young axolotls, the skeleton is cartilaginous, and with time it ossifies. Old axolotl femur is calcified all over the bone surface (yellow). In **a**, **b**: yellow - mineralized tissue, green - cartilage, light blue - connective tissue and red/orange is muscle. Scale bars 500 µm. **b** Femur epiphyseal ends of 6 cm (left), 13 cm (middle left), 25 cm (middle right) snout-to-tailtip axolotls and mouse tibia (right). Unlike mammals, amphibians do not have articular joints. The bone ends are cartilaginous and responsible for the length growth of the bone, analogous to the growth plate in the mouse. Scale bars 200 µm. **c** Axolotl epiphyses resemble the prenatal (E14.5) and early postnatal (P0) mouse bones. Movat’s pentachrome staining, or DAPI/EdU staining; scale bars 100 µm (left), 200 µm (right). **d** Safranin O/Light green staining of axolotl and mouse bones (cells are purple-brown, bone and soft tissue - green, cartilage - bright red). Left: bone marrow of an old axolotl. Note the huge fatty vacuoles (marked with * in **a**, **b**, **d**) and only few cells and not fully calcified bone cortices even in the old axolotls. Middle: bone marrow of 2 years-old mouse. Right: bone marrow of a young 12 weeks old mouse with only few fatty vacuoles. Scale bars 50 µm.

In order to understand axolotl bone healing and compare it to limb regeneration, a reliable fracture system is needed. In contrast to blastema, bone healing in amphibians, including axolotl, is hardly investigated^23^. Depending on the animal age and size, alcian blue-stained cartilaginous callus was observed in the small fracture site 20–45 days post-fracture^19,20,24^. Mitogawa et al. (2015) compared blastema and bone fracture healing and showed expression of Collagen I and Collagen II mRNA in the fracture region^24^. Based on this data, axolotl bone regeneration was assumed to resemble that of mammals, but no standardized fracture system was developed yet. Mostly, zeugopod bones (ulna and radius or tibia and fibula) were used, where one of the bones was cut with microsurgical scissors, and the other bone served as a limb fixator and supported the fractured bone^19,20,22,24^. However, in such a system, the fragments of the fractured bone can be misaligned and due to spontaneous bending of supporting bone often come in close proximity to the periosteum of the supporting bone^20^. This may lead to faster and inappropriate bone healing and wrong interpretation of the experiment results. Moreover, due to the difference in size and age of the animals used (which varied between 3 months and 1 year, and snout-to-tailtip size between 3 and 15 cm), there was no consistency in fracture gap size and healing dynamics across the animals in different laboratories. Besides, the bone diaphyses are not completely ossified in axolotls and other salamanders of this size range, and can vary from cartilaginous to partially ossified (Fig. 1a^25,26^), making the comparison of these studies difficult. Small fractures were mostly used as a healing-capable fracture control, and were created by simple mid-diaphyseal cut with iridectomy scissors. Several studies were performed using such small fracture setup: for instance, a simple cut of the ulna was repaired within 7 months^20^, the osteotomy in the fibula healed within 2 months, and a 10–20% bone length gap of a fibula (still defined as not critical) healed within 3 months^22^. Hutchinson et al. (2007) investigated the bone healing in young animals with a snout-to-tailtip length of 3–5 cm^20^ whereas others used young adult animals with a snout-to-tailtip size of 12–15 cm^22^ or of 8–12 cm^27^. Cosden-Decker et al. (2012) took 7–12 months old animals without stating the size^19^. In many of these studies, the critical size defect (CSD) non-union was described. Again, the CSD size and conditions were dependent on the size and age of the animals used: 2 mm radius defect was used in 8–12 cm axolotls^27^; 4 mm tibia defect in 7–12 months old animals^19^; 4 mm radius or tibia CSD in 3–5 cm long axolotls^20^; 2.8 mm, and 3.5 mm CSD of 7 mm-long fibula in 12–15 cm axolotls^22^. Due to indirect fixation by the neighbor bone, the fragments of the broken bone were fusing to the supporting bone in some cases. To be able to compare blastema and bone fracture, systemic characterization of standardized fracture is necessary. Moreover, to be able to extrapolate the results, an axolotl fracture model comparable to mammalian is needed.

For mammals, in particular for mice and rats, commonly used as fracture model organisms, various fixation methods are available, such as intramedullary pins, external fixators or internal bone-aligning plates^28,29^. Intramedullary pins provide bone stability, but a substantial part of the bone marrow is removed and pins may be difficult to install in the axolotl limb. External and internal plate fixators are suitable for a wide range of bones, and are less invasive to bone marrow. Since axolotl is an aquatic animal, internal plates seem to be preferable, because they are covered with soft tissues, such as muscle and skin.

In the present study, we show an application of a rigidly stabilized femur osteotomy model in axolotl and compare it to amputated limb regeneration as well as to mouse femur fracture healing. This load-bearing bone fracture model could prove useful for studying fracture healing in axolotl and investigating the possible ways of improving bone healing.

## Results

### Comparison of bone structure in young versus old axolotl and axolotl versus mouse

To compare bone healing in axolotl and mouse we first analyzed the bone structure of both species. Initially, during development the limb skeleton is cartilaginous in both axolotl (up to 4 cm snout-to-tailtip larvae) and mouse (until E15.5 in the hind limb^30^). In small 6 cm snout-to-tailtip axolotl larvaes the bone collar starts to calcify^26^ (Supplementary Fig. 1), in 13 cm snout-to-tailtip axolotls the epiphyses consisting of cartilage cells are longer in relation to the total bone length than in 25 cm, 5-8 years old axolotls (Fig. 1a, b). Similar to mouse, the bone diaphysis in old axolotls is calcified and contains a large bone cavity surrounded by thick cortical bone. Notably, even at the age of 5–8 years, axolotl seem not to have developed the secondary ossification centers yet.

Postnatally, long bones of mammalian appendages grow by perichondral/periosteal and endochondral replacement. The latter takes place at the epiphyses where the cartilaginous growth plate is located (Fig. 1a, b, right image “12 w.o. mouse”, in green) and contributes to the bone length increase. Similar to mammals, we found that the axolotl epiphysis contains five zones, namely the periarticular joint chondrocyte layer, followed by resting zone chondrocytes, then a layer of less mature EdU^+^ proliferating chondrocytes with columnar structure, transitioning closer to the bone center to a pre-hypertrophic and hypertrophic chondrocyte area (Fig. 1c). This resembles the structure of long bones in embryonic (E14,5) and early postnatal mouse (P0). Despite the different joint structure, the axolotl femur epiphysis resembles the mammalian growth plate and could contribute to the bone growth by endochondral ossification.

Interestingly, axolotls of 2 different sizes (“young”, 13 cm snout-to-tailtip body length, or “old”, ≥20 cm) both have bone marrow full of fatty cells (Fig. 1b, d). The bone marrow cavity in old axolotls is full of large fatty vacuoles with only very few cells in between (Fig. 1d). This is similar to old mice (2 years old) where bone marrow contains more fat than the bone marrow of young 12 week old mice. However, the bone cavity of aged mice still shows bone marrow cells other than adipocytes while in older axolotls the bone marrow cavity is filled completely with fatty vacuoles of adipocytes (Fig. 1d, Supplementary Fig. 2).

### Plate fixation can be applied to axolotl femur prior to fracture surgery

**Fig. 2:**
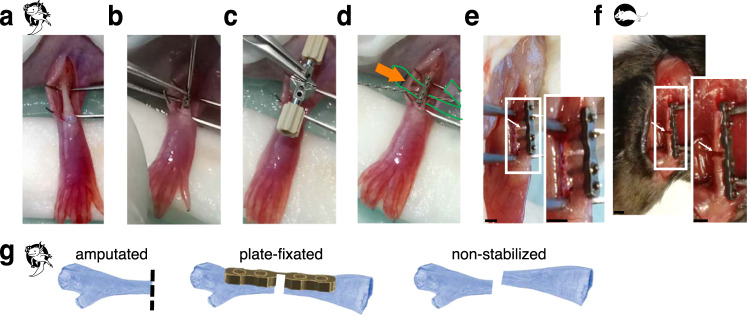
Overview of the surgical procedure and the experimental setup. **a** ≥20 cm snout-to-tailtip axolotl femurs were exposed by dissecting upper hind limb skin and spreading the muscles above the femur. **b** A titanium plate (7.75 mm Plate, RISystem, Switzerland) was attached to the femur by 4 titanium screws. **c** A saw guide was used for osteotomy. **d** A Gigly wire (0.66 mm) was inserted under the bone and used for sawing. Green outline and arrow show the plastic foil used for soft tissue protection during the surgery. **e** Osteotomy resulted in 0.7 mm bone gap in axolotl (white arrow). **f** 0.7 mm fracture of mouse femur is shown (white arrow). Here, a 6-hole plate (10 mm MouseFix Plate XL, RISystem) was attached to the femur by 4 titanium screws to fixate the bone. Scale bars 1 mm. **g** Sketch of the three different osteotomy models used. Amputation of the limb at the mid-femur (left) leads to blastema formation in the region of the proximal femur end of the osteotomy gap of the plate-fixated fracture (middle) and the non-stabilized fracture (right). In the non-stabilized fracture the two bone ends do not stay aligned but converge in a random way. Dashed line shows the amputation plane.

We aimed to establish a stable bone fixation method in the axolotl upper limb, preferably with a clinically relevant technique. One of the widely used bone fixation methods is an internal plate fixation, which provides mechanically rigid stabilization of the femur (Fig. 2). In 5-8 years old, ≥20 cm snout-to-tailtip long axolotls, the femur resembles in size and shape that of a murine femur. In these animals the femur exceeds 1 cm and is wide enough to apply a plate fixator (7.75 mm MouseFix Plate, RISystem). Thus we decided to test plate fixator application on the axolotl femur and characterize the healing process of the 0.7 mm osteotomy, which was shown to heal via endochondral ossification in mouse^31^. The surgery summary is shown in Fig. 2. Notably, the axolotl femur narrows towards the bone center, and the middle of the plate fixator was aligned with the region of the bone with the smallest diameter. The axolotl femur is more fragile than that of the mouse, so care should be taken during the drilling step, followed by the plate fixation using the titanium screws (Fig. 2b). Besides, bone wax application proved to be useful (Supplementary Fig. 3), since axolotls do not have large muscles able to cover the plate preventing screw heads to scratch the skin from inside.

As a comparative example of successful bone regeneration in aged axolotls we used limb amputation (Fig. 2g, left) at the proximal osteotomy side in order to compare bone healing and regenerative capacities in aged axolotl. Notably, the limb regeneration is slower in the large axolotls, therefore, more time was given to the animals to regenerate prior to sample harvesting. To evaluate if the bone healing potential is increased in the plate-fixated limbs, we used non-stabilized fracture as a negative control (Fig. 2g, right). In case of non-stabilized fracture we expected a higher mobility of the bone fragments, leading to abnormal bone healing.

### Plate-fixated femurs show smaller callus formation and better fracture healing

**Fig. 3:**
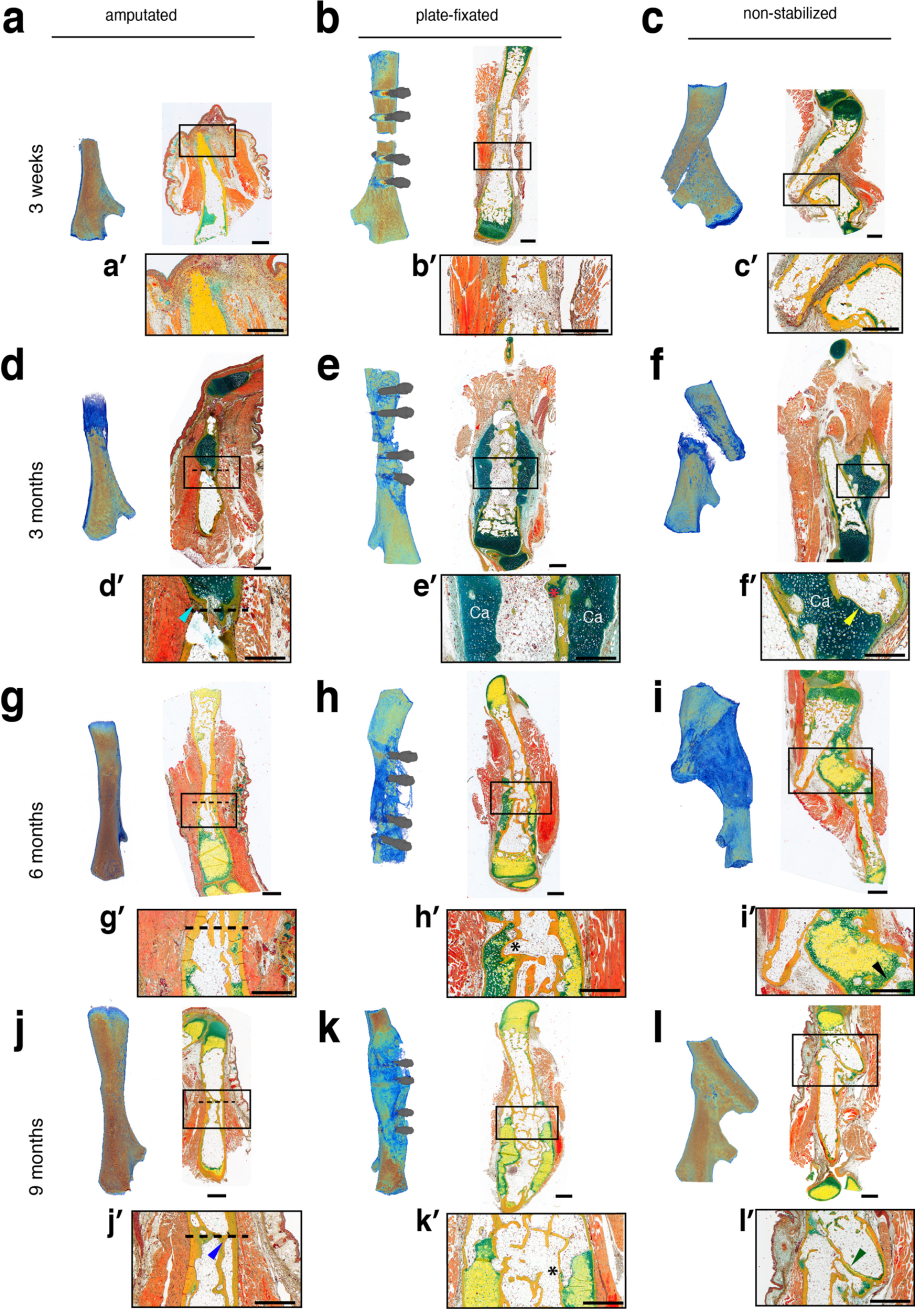
Plate stabilization retains bone alignment and leads to smaller callus formation with quicker transition to woven bone. Amputated upper hind limb (left), plate-fixated (middle), and non-fixated (right) femur fracture 3 weeks (**a**–**c**), 3 (**d**–**f**), 6 (**g**–**i**) and 9 (**j**–**l**) months after bone injury. Micro-CT analysis (left panels) and tissue sections stained after Movat’s pentachrome protocol (right panels) are shown (mineralized tissue=yellow; cartilage=green, muscle=orange). **a** 3 weeks post-amputation, a regeneration blastema is formed at the tip of the leg stump. **b** In plate-fixated sample, fracture gap is clearly visible at micro-CT image. **c**. The non-fixated samples show severe bone misalignment. **d** Blastema at 3 months post-amputation. Dashed line – amputation plane in **d**, **g**, **j**. **e** Stable fixation with the bone-aligning plate provides stability and allows for formation of a cartilage sleeve bridging the bone gap. Note incomplete cortex beyond the fracture gap on the right side (red asterisk). **f** In non-fixated fracture, a cartilage front formed but the bone fragments are not aligned in the proper axis. The medullary cavity sealed on both sides of the fracture gap, indicating terminated bone formation at the fracture ends (**f**’, yellow arrowhead). **g** 6 months post-amputation, skeletal elements re-grow. The amputation plane—dashed line. **h** Plate-fixated bone improves the cortical thickness and stability by growing a second cortex (black asterisk in **h**’, **k**’). **i** Delayed mineralization and longer persistence of cartilage in the callus of the non-fixated fracture. **j** 9 months post-amputation, limb is fully regenerated (**j**’, blue arrowhead—secondary cortex). **k** Plate-fixated samples 9 months post-fracture with developed ossified hard callus, secondary cortices and fat vacuoles-filled bone cavity. **l** The non-stabilized fracture achieved bony bridging of the gap—the osteotomy ends of the bone showed a rounded appearance (**l**’, green arrowhead). Scale bars 1 mm.

To assess fracture healing and limb regeneration in aged axolotls, we harvested samples 3 weeks, and 3, 6 and 9 months after osteotomy or amputation. While an osteotomy gap of 0.7 mm (non-critical) in mice is bridged within 3–4 weeks^31,32^ (Supplementary Fig. 4), surprisingly no sign of bone formation was observed in axolotl fracture samples analyzed by micro-CT or histology 3 weeks post-osteotomy, and the fracture gap was filled with cells and dense ECM (Fig. 3b,c). Bone fragments were well-aligned in the plate-fixated samples (Fig. 3b, *n* = 4/6), in contrast to severe non-fixated bones misalignment (Fig. 3c, *n* = 7/7). Histological staining after Movat’s protocol shows accumulation of cells and ECM in the fracture gap in the plate-fixated and non-stabilized samples. The delay in bone bridging in axolotl was unexpected, since in the 3 weeks post-amputation blastema a cartilaginous stump callus was already formed (Fig. 3a, *n* = 2/2).

Already 3 months after amputation, axolotls had regenerated a newly patterned limb that contains both soft tissues and mostly cartilaginous skeleton of the hind limb, including distal parts of femur, tibia and fibula, metatarsus and digits (Fig. 3d, *n* = 2/2). Proximal to the amputation plane, the newly regenerating bone started to mineralize. Histological images showed a clear border between the pre-amputation “old” bone which is filled with fatty vacuoles and only few cells, and the distal regrown bone (cyan arrowhead in Fig. 3d’). Outside the more distal cartilaginous skeleton elements the cortices are forming.

3 months after surgery of plate-fixated bone, fragments remained aligned to the bone axis, retaining the bone length, and the fracture is nearly bridged (Fig. 3e), whereas non-fixated bone fragments are misaligned and overlap, and the limb length is not retained (Fig. 3f). The micro-CT analysis indicated mineralized tissue within the fracture gap of plate-fixated femurs. Histology revealed cartilaginous soft callus formation in the fracture area with partial, although incomplete bone bridging (*n* = 3/4). In the plate-fixated fracture samples, we observed cartilage connections between the cortical bone ends and a partial reconnection of the mineralized cortices (Fig. 3e’, red asterisk).

In contrast, in the non-fixated bone no mineralized bridging could be detected after 3 months of healing. Here, the bone ends were not aligned (5/5) and moreover, in 2 out of 5 samples the misalignmet was so severe that the proximal fragment of the femur touched the side of the distal fragment. A cartilaginous callus is formed, but it is only weakly mineralized at 3 months post-fracture (Fig. 3f, *n* = 4/5). Interestingly, the non-fixated osteotomy revealed closing bone fragment ends indicating starting bone formation in this area (yellow arrowhead, Fig. 3f’). In the areas where bone fragments came in close proximity, a cartilaginous bridge was formed. Notably, the callus is formed between the edge of fractured bone and the intact periosteum of the opposite bone fragment, forming a completely new bone continuity (Ca, Fig. 3f’, Supplementary Fig. 9).

6 months post-amputation all regenerated bones started ossifying, however the length of the bones is not yet comparable to that of uninjured limb (Fig. 3g, *n* = 2/2). Less cartilage compared to the earlier time point and more mature bone was observed. The bone marrow was filled with fatty vacuoles with only few cells, and the cortices were solid. No difference between the bone marrow proximal and distal to the amputation was detectable any more (Fig. 3g, amputation plane still noticeable due to the presence of dense cortical network, dashed line).

After 6 months of healing the plate-fixated fracture showed complete bony bridging (Fig. 3h, *n* = 4/4). The gap fully bridged and the cartilage callus is ossifying, secondary cortex formed to gain the bone stability (Fig. 3h’, black asterisk).

In the non-stabilized fracture the gap between the proximal part of the fracture and the side of the distal bone fragment was bridged with ossifying callus but had a thinner cortical diameter compared to the other bone parts (Fig. 3i, *n* = 4/4). The length difference of the femur was still clearly visible with the original length only being maintained with stable fixation.

9 months after the amputation the femur is fully regenerated in size and structure (*n* = 3/3). The amputation plane was only manifested by denser network of cortical bone fragments, indicating remodeling of the bone (Fig. 3j’, blue arrow). The plate-fixated fracture gap was fully bridged with bone (Fig. 3k, *n* = 3/3). Notably, the former soft callus is replaced with hard callus, filled with characteristic to axolotl fatty vacuoles in the bone cavity (black asterisk). The hard callus formation is known in the mouse bone healing as well.

After 9 months of healing the non-fixated bone fracture remarkably completed bony bridging (green arrow) and restored the normal cortical thickness (Fig. 3l). Only remnants of cartilage are still present. However, the non-fixated fracture was not able to regain the normal femur length (*n* = 3/3).

In conclusion, micro-CT and histological analysis showed efficient restoration of the bone length and structure upon amputation. In comparison to the non-fixated femur, plate-fixated bone healing upon osteotomy regains bone integrity more efficiently. This may be explained by necessity to produce larger callus in non-fixated femur to compensate for the bone fragments misalignment. It is remarkable that bridging of a non-critical gap seems to be more difficult for axolotl than re-growing the complete distal limb, which was achieved in the same time frame.

### Callus in mouse and axolotl contains SOX9^+^ cells (cartilage)

**Fig. 4:**
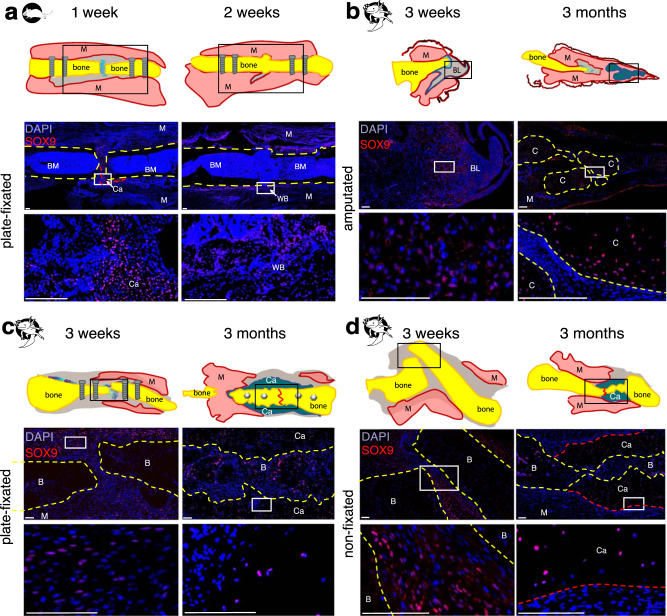
Comparison of SOX9 expression in fractured bones and amputated limbs. **a** In axolotl amputated limbs, SOX9-positive cells are present in 3 weeks blastema and cartilaginous condensations in 3 months regenerate. **b, c** In axolotl fixated and non-stabilized fractures, SOX9 expression is observed in pre-cartilage cells at 3 weeks post-fracture and in callus cells at 3 months post-fracture. **d** In murine fractures, SOX9-positive cells are present at 1 week post-fracture, and at 2 weeks, bone bridging by woven bone is in progress. Scale bars 200 µm. Yellow dashed line - bone, red dashed line - callus, BM – bone marrow, M – muscle, C – cartilage, Ca – callus, BL – blastema, B – bone.

Mammalian long bone fracture healing goes through the process of endochondral ossification, recapitulating the developmental process when cartilaginous template is gradually substituted by the ossified bone tissue. In this process, SOX9-positive cartilage progenitor cells are accumulating in and around the fracture gap, forming a callus. In mouse, SOX9-expressing cells were observed in the fracture site already at 1 week post-surgery, and further accumulate during the fracture callus formation. Notably, this process appears to take place in the similar time range in aged (Fig. 4a) and young (Supplementary Fig. 5) mice. A mouse osteotomy is bridged at 2 weeks post-surgery, when woven bone is connecting the bone fragments over the fracture gap (Fig. 4a, Supplementary Figs. 4, 5).

To check if the SOX9-positive cartilage progenitors accumulate in axolotl fracture healing and axolotl limb regeneration, we used immunofluorescent staining for SOX9. In regenerating blastema SOX9-positive cells appeared already at 3 weeks post-surgery prior to cartilage formation and later comprised the majority of cells in the cartilaginous skeletal elements of newly-forming limb (Fig. 4b). Interestingly, even in ossifying bones at later regeneration stages (6 and 9 months post-amputation) SOX9-positive cells remained abundant (Supplementary Fig. 6).

Remarkably, although the Movat Pentachrome staining did not show the characteristic cartilage pattern at 3 weeks post-fracture, the fluorescent staining for SOX9 showed accumulation of cartilage progenitors in both plate-fixated and non-stabilized fractures. SOX9 expression was observed in the cartilaginous callus at 3 months post-fracture (Fig. 4c, d), lowering at 6 months post-fracture until almost disappearing at 9 months post-fracture (Supplementary Fig. 5). Decrease in the SOX9 expression coincides with the callus maturation and ossification supporting the endochondral ossification as mechanism of bone fracture healing in axolotl.

### Proliferation precedes callus formation in axolotl and mouse

**Fig. 5:**
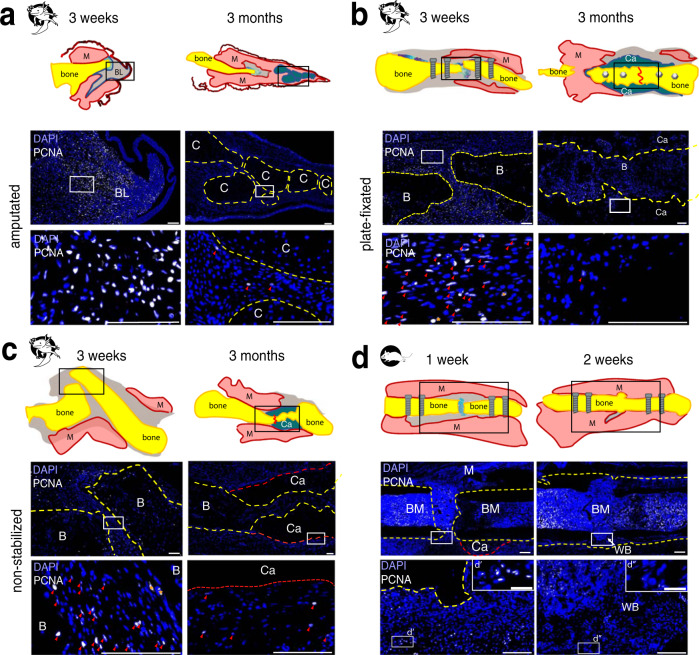
PCNA immunostaining showed a decreased proliferation in amputated limbs and fractured bones at 3 months post-surgery. Representative samples of axolotl upper hind limbs stained with anti-PCNA (white) and DAPI (blue) 3 weeks and 3 months post-surgery in axolotl amputated limbs (**a**), in plate-fixated fractures (**b**), and in fractures without fixator (**c**). Note high number of PCNA^+^ cells in 3 weeks axolotl samples. Only a few PCNA^+^ cells can be observed in 3 months samples. Here, proliferating cells are mostly observed in the soft tissues. **d** Mouse fracture samples show many PCNA^+^ cells in the proximity to injury site at 1 week post-fracture. At 2 weeks post-fracture, PCNA^+^ cells are retained in the bone marrow, and not in the newly formed bone. d’ and d” show enlarged boxed areas. Scale bars 200 µm, 50 µm in d’ and d”. Yellow dashed line - bone, red dashed line - callus, red line on sketches – former fracture site, BM – bone marrow, M – muscle, C – cartilage, Ca – callus, BL – blastema, B – bone, red arrows show PCNA^+^ cells.

In the amputated axolotl limb, cells from surrounding tissue de-differentiate, migrate towards the amputation plane and form a blastema. Limb blastema formation coincides with the active proliferation phase (shown by PCNA, Proliferating Cell Nuclear Antigen, expression, Fig. 5a). Notably, at 3 weeks post-fracture, we observed PCNA-positive cells in both plate-fixated and non-stabilized fractures (Fig. 5b, c). At 3 months post-fracture we observed a strong reduction in cell proliferation in fractured bones and surrounding tissue (Fig. 5b, c), retained at 6 and 9 months post-surgery (Supplementary Fig. 7). Proliferating cells were mostly located in the soft tissues, surrounding the cartilaginous callus.

In mouse, we observed PCNA^+^ cell accumulation at 1 week post-fracture in both 1 year-old (Fig. 5d) and 12 weeks-old (Supplementary Fig. 8) mice. 2 weeks post-fracture, there are only a few PCNA^+^ cells visible in the fracture gap, although the bone marrow shows numerous PCNA^+^ cells.

## Discussion

Axolotls have a high regenerative capacity for whole limb regeneration after amputation. Already in 2006 Roy and Lévesque concluded that the regeneration capacities of axolotls and mammals are different not because of unique molecular pathways used in axolotls that are absent in mammals or vice versa^33^. It rather seems that they are linked to the way these pathways are activated and modulated in response to wounding. Since bone can heal without scar formation in both mammals and salamanders, it represents an interesting tissue for regeneration research and the axolotl may offer important insights on why the efforts to stimulate human regeneration have been paved with difficulties^12,34,35^. Moreover, recent development of transgenesis and efficient knock-out methods, baculovirus and retrovirus overexpression systems, fluorescent in situ hybridization technique, and deciphering of genome and transcriptome places the axolotl in an advantageous position among the regenerative model organisms^36–44^. However, no standardized bone fracture model existed for axolotl, making it more difficult to perform comparable studies. The aim of this study was to establish in axolotl a controllable bone fixation system analogous to mammals.

To be able to compare the fracture healing in axolotl and mouse, we investigated the morphological properties of the developing axolotl bones and compared them to mouse. Surprisingly, despite being a widely used model of appendage regeneration, axolotl limb skeleton has not been described in details until recently. A first glance at the bone and joint development was taken by Cosden-Decker et al. (2012) with an emphasis on femorotibial joint development^19^. Here, the epiphyseal cartilage zone adjacent to the medullary cavity was described in axolotls aged 2-24 months. We also saw the epiphyseal parts of axolotl femur resembling the mammalian growth plate, indicating endochondral longitudinal growth of axolotl bones. Moreover, we show that the axolotl metaphysis shows a distinct proliferation zone, resembling the growth plate of mammalian bones. *Pleurodeles waltl*, another urodele amphibian, was also shown to have epiphyses with similar structure^25^. Interestingly, Cosden-Decker et al. (2012) also observed reduction in the cartilage zone size in comparison to the bone length in larger animals, but gave no description of the diaphyseal part of the bone^19^. Here, we show that the diaphyses of axolotl are gradually ossifying, although slower than in the mouse. At the age of 5-8 years axolotls still lack the secondary ossification centers, similar to the other salamanders^25,45^. The reason could be the aquatic nature of axolotl and the fact that the analyzed animals did not undergo metamorphosis, i.e. even the aged axolotls stayed aquatic. However, the bone marrow of ossified axolotl bones contained numerous fatty vacuoles with rare cells in between. A similar observation was made by Riquelme-Guzman et al. (2021)^26^. We observed comparable morphology in the long bones of aged mice. Previously, an absence of blood cell-generating CFUs (colony-forming units) was shown in the bone marrow of axolotl^46,47^. This can be explained by the low number of cells in general, and the predominance of fatty vacuoles in the bone marrow cavity, observed already in the early ossifying axolotl bones. Another difference between terrestrial mammals and the aquatic axolotls is the load-bearing aspect of their long bones in consequence of their differences in ambulation. The biomechanical environment for locomotion on land and in water is different and could affect the healing process. Biomechanical stimuli are beneficial for bone healing if they are neither too low or too high^48^. However in case of the fixated bone of axolotl and mice in this study running and swimming (higher muscle forces) should provide the biomechanical stimulus that enables bone formation. In mice this is supported by the consolidated bone 3 weeks after osteotomy. That the mechanical stimulus is present in axolotl as well is supported by the fact that bone of the axolotl was able to form a callus. Age and gap size also determine the biomechanical influence during bone healing^49–51^, factors we tried to minimize within this study by using aged animals in both spezies and causing an equal-sized bone defect.

In the present study, we used 5-8 years old aged axolotls with a snout-to-tailtip length of ≥20 cm, where the femur diaphysis is fully ossified and is comparable by size to the mouse femur. We established a controlled fixation of a standardized fracture by stabilizing the femur with a plate fixator, which can be easily applied in the large axolotls. Additionally, the device comes in different types making it possible to study different mechanical conditions such as rigid and semirigid fixation or suitable for x-ray and micro-CT examinations (in this study, we used the rigid plate as fixator). It makes axolotl bone healing studies analogous to mouse, allowing for direct comparison of the cellular and molecular events. Moreover, it corresponds to the common standard of clinically used medical devices. In the past, all studies of axolotl fracture healing were done when one of the two zeugopod bones was fractured while the other was kept intact and served as stabilization^19,20,23,27^. Despite the supporting function the stabilizing bone could still bend and did not provide standardized gap size and full bone alignment. In the present study, we compared the plate-fixated bones to non-stabilized fractures and demonstrated better healing when a stable fixation was used compared to the non-fixated bone. The non-fixated bone was not aligned and it bridged at random connecting parts of the femur coupled with limb shortening, causing formation of a larger callus and requiring longer time to heal. This phenomena is observed in the displaced bone fractures in mammals, including human^52,53^. Therefore, plate fixation was expected to improve the bone alignment and make the bone healing studies comparable in the axolotls to mammalian bone healing.

In this study, we examined the axolotl bone healing creating a 0.7 mm gap, stabilized by plate fixator. Since the axolotl femur is similar in size and shape to mouse, we expected bridging of the fracture gap at 2–3 weeks post-fracture. Moreover, previous studies of axolotl articular cartilage defect healing have shown chondrocytic cell accumulation at 2–4 weeks post-surgery and partial cartilage restoration by 3 months^21^. Surprisingly, in the presented fracture model not only we did not observe the gap closure by 3 weeks, but even the callus tissue was not formed. Indeed, in the animals analyzed, the fracture healing is much delayed and takes 6–9 months to form ossifying callus. In mouse, a fracture of the same size (0.7 mm) stabilized with the plate fixation is bridged within 3 weeks^31^, and even in aged mice is bridged within 3–4 weeks^32^. In our experiments, we also observed bone bridging at 2 weeks post-fracture both in young and 1 year-old mice. The delay of axolotl bone healing could be explained by the slower cell proliferation rates in axolotls (average cell cycle is 44–60 hours^54^ compared to mouse cell cycle of 14–20 h^55^), age of axolotls and the characteristic morphology of the bone marrow cavity, resembling that of the aged mouse.

To be able to compare the axolotl amputated limb and fracture regeneration to the bone healing in mammals, we should define if the latter is recapitulating the progenitor cells accumulation and proliferation, and if the endochondral ossification is taking place in axolotls. The migration of SOX9^+^ periosteal cells was shown in the early fracture and soft callus and was shown to be part of the endochondral ossification process in mammalian fracture repair^56,57^. In the mouse samples, we observe accumulation of SOX9^+^ cells in the fracture gap at 1 week post-fracture, prior to cartilage condensation, coinciding with accumulation of PCNA^+^ proliferating cells. By 2 weeks post-fracture, fracture gap is filled with the woven bone and proliferation is reduced. In current study, the peak of cell proliferation in axolotl happened at the mid-blastema stage, and 3 weeks post-fracture, and already at 3 months ceases to low levels and is mostly retained in the soft tissues. In the axolotl samples, SOX9^+^ cells are already present at 3 weeks fracture samples, also in the 3 weeks post-amputation limb blastema despite the absence of the cartilage condensations. In case of murine and axolotl fracture, this quick proliferation can be a prerequisite for the callus formation. The morphologically cartilaginous fracture callus is present in 3 months post-surgery samples in axolotl. Once cartilaginous callus is formed, the proliferation is diminished and re-organization of callus into bone is taking place, similar to murine fracture healing study by Li et al.^58^. Interestingly, the skeleton of the axolotl regenerating limb was shown to be formed by progenitor cells originating from the periskeleton and soft connective tissue of the stump^17,59,60^. Therefore, whether the callus of axolotl fracture was formed from pre-existing SOX9^+^ periosteal cells or from cells expressing SOX9 de novo, is yet to be defined.

In conclusion, the here presented plate-fixated fracture surgery model in axolotl enables controlled and comparable investigation of bone healing across amphibians and mammals and direct comparison to the axolotl limb regeneration. This could be a first step to determine where blastema progression in amputated limbs and callus formation during bone healing deviate from each other and how this compares to the mammal situation in order to harness this knowledge to improve fracture healing in the future.

## Methods

### Experimental design

Axolotls (*Ambystoma mexicanum*) were bred in the Research Institute of Molecular Pathology facility. All animal handling and experimental procedures were carried out as approved by the Magistrate of Vienna (GZ: 51072/2019/16, GZ 65248-2021-26). 5–8 years old, ≥ 20 cm snout-to-tailtip (snout to the tip of the tail) long axolotls, were used for the fracture surgery and amputations.

C57BL/6 J mice were bred at the Comparative Medicine Facility of Research Institute of Molecular Pathology, were kept at a 12‐h light/dark cycle and were fed a standard diet with water *ad libitum*. All animal procedures were performed according to the Austrian animal welfare act guidelines for the use of experimental animals and were approved by the Magistrate of Vienna (GZ: 144706/2018/18). 12–14 weeks old female mice and 56-61 weeks old female and male mice were used for left hind limb femur surgery.

### Osteotomy in axolotl

The animals were anaesthetized in 0.03% benzocaine solution (Sigma–Aldrich, Germany) for about 15–20 min until good muscle relaxation and lack of reflexive movement upon limb touching with tweezers. Then the animal was placed on wet paper towels soaked in 0.03% benzocaine solution and covered with benzocaine-soaked paper towels. A hindlimb was stretched and a longitudinal incision was made above the femur bone in the upper hind limb. The muscles and nerves were carefully displaced from the surgery site without cutting using bowed forceps. The femur was gently lifted by using bowed forceps and thus exposed to the surgery. To prevent soft tissue damage, a piece of plastic film was placed under the femur. The comparable size of femur in aged axolotl and mouse enabled us to use a commercially available rigid 7.75 mm 4-hole fixator plate (MouseFix Plate, RISystem, Switzerland).

The MouseFix Plate was aligned with the femur surface and placed avoiding touching the joints. Four 2 mm titanium screws were used to attach the bone to the plate. To assure the plate being parallel to the bone axis, it is crucial to mount the inner screws first, and then the two outer screws. Next, saw-guiding device was temporarily attached to the screws, and 0.66 mm Gigly wire saw (RISystem, Switzerland) was used to make a single 0.7 mm cut in the femur. Constant irrigation with 0.7x PBS was used during drilling and sawing to minimize tissue damage. After bone was cut, saw, saw guide and protection film were removed, and the screws were covered with a sterile bone wax (Ethicon, USA) to protect skin and muscles from irritation by screws. Then, muscles were placed back and skin was stitched using 7.0 Optilene suture (Braun, Germany) (Fig. 2).

To create a non-fixed femur fracture the bone was prepared as in the osteotomy surgery. The femur was then cut using 8 cm iridectomy scissors (blade size 1 cm) in the same mid shaft position as the gap in the fixated bones. The muscles were placed back and skin was closed with single stitches using 7.0 Optilene suture (Braun, Germany).

For creating blastema sample, hind limb was amputated at the midshaft of the femur diaphysis using a scalpel blade. To prevent an irregular shape of the blastema, the femur was trimmed using iridectomy scissors to match the level of surrounding tissue. The amputation was performed so that the resulting stump resembled the proximal bone end of the osteotomized bone to compare the regeneration with the bone healing process—to have the same proximal bone end in all three groups (Fig. 2).

After the bone fracture surgery, animals were kept for 3 days in artificial pond water with 50 U/mL penicillin und 20 µg/mL streptomycin (Gibco, 15140-122). Carprofen (Rimadyl, 5 mg/L of pond water) was used as pain medication for 3 days post-surgery.

### Osteotomy in mouse

Femur osteotomy was performed in 12–14 weeks old female mice. In short, animals received s.c. buprenorphine injection (0.1 mg/kg) (Richter Pharma AG, Austria) and enrofloxacin (Baytril, Bayer, Germany) prior to the surgery. Isoflurane (Piramal Healthcare, UK) and O_2_ supplementation were used for anesthesia. Gentamicin gel (Gentax, Slovakia) was applied to prevent eye drying. The left upper hind limb was shaved and disinfected with iodine solution (Betaisodona, Mundipharma, Austria). 6-hole MouseFix plates (RISystem, Switzerland) were used for bone fixation and 0.66 mm Gigly wire saw (RISystem, Switzerland) was used to create a femur osteotomy in mouse^6,61^. Constant irrigation with PBS and protection with plastic foil were used to minimize tissue damage. After the surgery, soft tissues and skin were placed over the stabilized bone, and skin was stitched with Novosyn 5.0 suture (BBraun, Germany). The skin was sprayed with a liquid wound spray (Hansaplast, Beiersdorf AG, Germany). Animals were provided with Tramadol (50 mg/l) (Grunenthal, Germany) for the first 3 days post-surgery via drinking water (changed daily). Animals were euthanized via cervical dislocation in deep anesthesia after 7, 14, and 28 days post-surgery respectively.

### Sample preparation

Axolotl limbs were harvested at 3 weeks, 3, 6, and 9 months post-surgery, the tissue was fixed in MEMFA buffer (10xMEM salt (1 M MOPS pH 7.4, 20 mM EGTA, 10 mM MgSO4), 3.7% formaldehyde in mQ water) for 24 hours at +6 °C and subsequently washed in PBS prior to further processing.

Mouse tissues were fixed in 4%PFA in PBS for 24 h at +6 °C, washed in PBS, de-calcified in 0.5 M EDTA for 5 days, +6 °C, and treated with 30% sucrose/PBS solution prior to embedding in TissueTek OCT (Science Service, Germany).

### Microcomputer tomography of axolotl limbs

Fixed axolotl bones underwent microcomputer tomography in a custom-made holder prior to histological embedding. Bones were scanned with a nominal resolution of 10.05 µm in a Bruker SkyScan 1172 (Billerica, MA, USA) high-resolution micro-CT. A 0.5 mm aluminum filter was employed and an x-ray tube voltage of 59 kV, 141 µA and maximized power. Image acquisition was done with a 180° orbital in 0.3° rotation step and camera pixel binning for higher sensitivity. Reconstruction was carried out with a modified Feldkamp algorithm using the SkyScan NRecon software. Gaussian smoothing, ring artefact reduction, misalignment compensation and beam hardening correction were applied. Imaging was performed using SkyScan CTvox software.

### Whole-mount alcian blue/alizarin staining of axolotl limbs

For defining cartilage and calcified bone in axolotl limbs, the right fore- and hindlimbs were harvested from 6-6.5 cm snout-to-tailtip axoltl larvae and fixed in 4% PFA in PBS, pH 7.4 for 24 h, +6 °C and stained with alcian blue/alizarin red^20^. The limbs were washed in PBS, dehydrated in ascending ethanol solutions (25%, 50%, 75%) and then stained in 0.1 mg/mL alcian blue solution. Then, the limbs were rehydrated, washed in 1% KOH solution and subjected to 0.1 mg/mL alizarin red staining. Finally, the limbs were washed with 1% KOH, dehydrated and stored/imaged in glycerol.

### Histology

For histological analysis, axolotl bones were embedded in paraffin and 5 µm thick longitudinal sections were cut and stained with Movat’s Pentachrome or Safranin O/ Light green.

For Movat’s Pentachrome, tissue was deparaffinized and re-hydrated, and incubated for 3 min in 3% acetic acid, for 30 min in 1% Alcian Blue (Sigma Aldrich, Germany)/3% acetic acid, and differentiated for 3–5 min in 3% acetic acid. After washing in ddH2O, sections were incubated for 1 h in ammonia/ethanol, washed twice in tap water, shortly in ddH2O and stained in Weigert’s iron hematoxylin (Chroma-Waldeck, Germany) for 10 min. Sections were washed in tap water and incubated for 15 min in brilliant crocein R fuchsine (Chroma-Waldeck, Germany). After short rinse in 0.5% acidic acid, sections were incubated in 5% phosphotungstic acid (Chroma-Waldeck, Germany) for 20 min and 1 min in 0.5% acetic acid, washed 3 × 5 min in 100% EtOH and stained with Saffron-du-Gatinais (Chroma-Waldeck, Germany) for 1 h. After dehydrating 3 × 2 min in 100% EtOH, slides were washed in Xylol and embedded. Staining with Movat’s Pentachrome staining allows for discrimination of fibrous tissue (light green/blue), calcified bone (yellow), muscle (red) and cartilage (intensive green).

For Safranin O / Light green staining slides were rehydrated and stained in 1% safranin O (Merck, Germany) for 8 min. Afterwards the sections were dipped in ddH2O 5 times and placed in 0.1% Light green (Chroma-Waldeck, Germany) for 5 min. The sections were then dipped 5 times in 1% acidic acid followed by 2 times for 2 min 100% EtOH treatment. To finish the staining the sections were placed in Xylol 2 times for 2 min and embedded. Safranin O/Light green results in staining bone and soft tissue in green, cartilage -intensive red, and cells in purple/brown.

Movat Pentachrome staining on mouse samples was performed on 12 µm cryosections according to the axolotl protocol as described above, omitting deparaffinization step. Safranin O/Light green staining was done on mouse paraffin 5 µm sections or 12 µm cryosections accordingly to the protocol described above.

### Immunohistology

12 µm cryosections (mouse) and 5 µm paraffin sections (axolotl) were used for immunodetection. If necessary, antigen retrieval with DAKO solution (S1699, Agilent, USA) was used (Supplementary Table 1). For all antibodies, tissue sections were initially blocked with 5% BSA in PBS buffer with 0,1% Tween-20 for 1 hour prior to applying the primary antibody overnight, +6 °C. (Supplementary Table 1). Antigen retrieval method, dilution and incubation times for all primary antibodies are listed in the supplementary material, Supplementary Table 1. Secondary antibodies conjugated to Alexa fluorophores (Invitrogen) were applied at 1:500 dilution in PBS with 5% BSA with 0,1% Tween-20 for 2 h, RT. Stained slides were covered with Mowiol/DABCO.

Fluorescence images were captured with a Pannoramic Slide Scanner 250 Flash III (3D HISTECH, Hungary), processed in CaseViewer 2.2 software (3D HISTECH, Hungary) and then colored and merged in Fiji.

### EdU labeling in axolotl

11 cm snout-to-tailtip axolotls were injected with 10 microgram EdU per gram body weight every second day for 2 weeks. Upon harvesting, limbs were fixed in 4% PFA in PBS for 24 h at +6 °C, washed in PBS, de-calcified in 0.5 M EDTA for 2 days, +6 °C, and treated with 30% sucrose/PBS solution prior to embedding in TissueTek OCT (Science Service, Germany). 12 µm sections were stained with Click-iT EdU Cell Proliferation Kit (ThermoFirscher Scientific) and DAPI and scanned as described above.

### Reporting summary

Further information on research design is available in the Nature Research Reporting Summary linked to this article.

## Supplementary information

Supplementary figures April 2022

REPORTING SUMMARY

## Data Availability

The data that support the findings of this study are available from the corresponding author upon reasonable request.
