# Supplementary material for: The specialist in regeneration—the Axolotl—a suitable model to study bone healing?
Source: NPJ Regen Med. 2022 Jun 30;7:35. doi: 10.1038/s41536-022-00229-4 (PMC9246919; doi:10.1038/s41536-022-00229-4)
Supplement: Supplementary file 1 — Supplementary figures April 2022 [file 41536_2022_229_MOESM1_ESM.pdf]

Supplementary figures

Supplementary Figure 1. *Bone collars ossify in 6 cm snout-to-tailtip axolotls.*

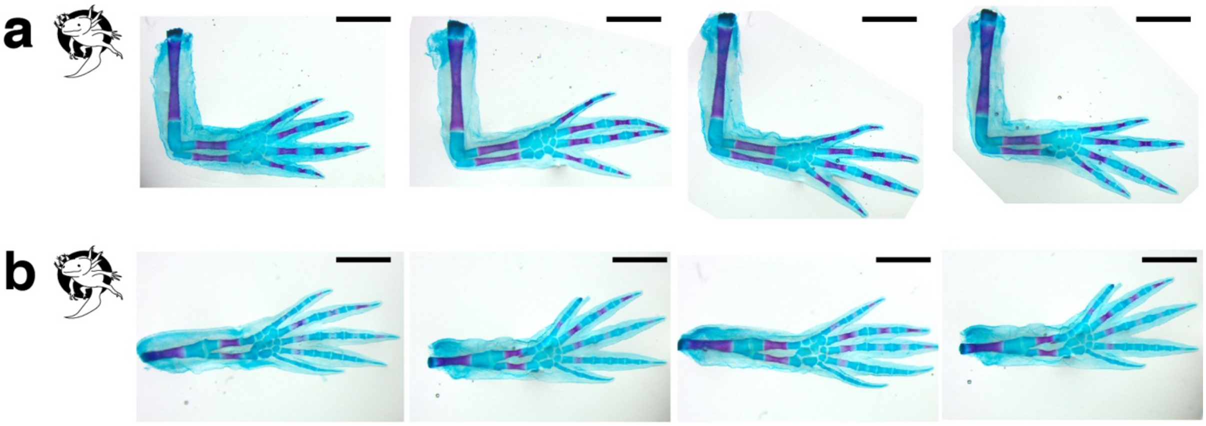

6 cm snout-to-tailtip axolotl fore- (**a**) and hindlimbs (**b**) were stained with alcian blue/alizarin red.

Scale bars 1mm.

Supplementary Figure 2. *Distribution of adipocytes in long bones of aged axolotl and mouse.*

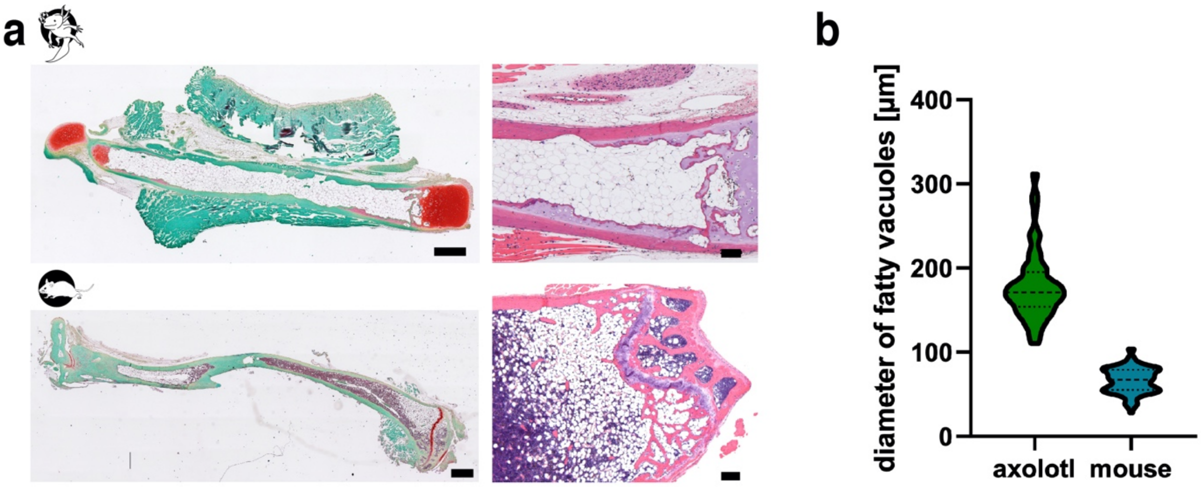

a. Left: In aged axolotl (femur) adipocytes with their fatty vacuoles fill out the bone marrow space completely while in mice (tibia) adipocytes are predominant at the bone ends and the mid-diaphyseal area still shows other bone marrow cells – depicted by the dark red area in the histological staining. Safranin O - Light green staining shows tissue in green and cartilage in bright red. Middle: In higher magnification the bone ends are depicted to show the difference in adipocyte size. HE staining: mineralized bone - red, nuclei - blue, cartilage – lila. **b.** 100 fatty vacuoles were measured at their widest point in mouse and axolotl and the diameter is depicted in µm in the graph. The fatty vacuoles of axolotl are significantly larger than those found in mice ($p<0.0001$, t-Test).

Supplementary Figure 3. *Extended surgery procedure.*

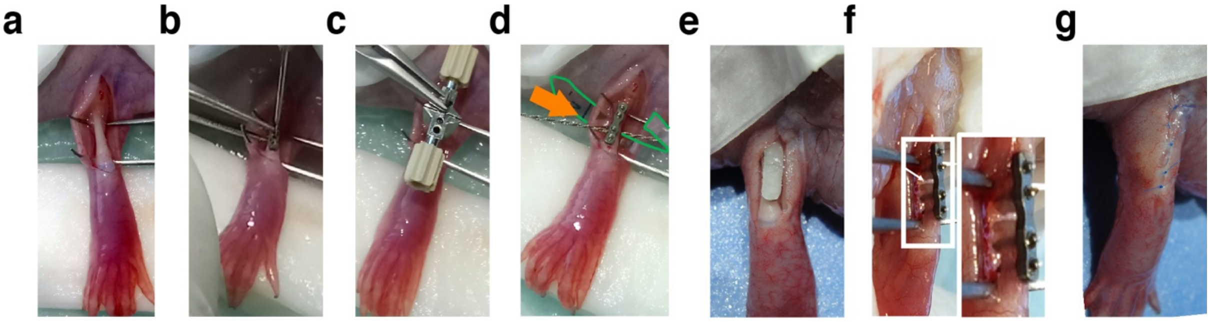

a. Femur was exposed for the surgery. **b.** A titanium plate was attached to the femur by 4 titanium screws. **c.** A saw guide was used for osteotomy. **d**. A Gigly wire (0.66 mm) was inserted under the bone and used for sawing. Note plastic foil usage for nerve and vessel protection during the cutting step (**d**) and bee wax application on top of the titanium screws (**e**). **f.** Osteotomy resulted in 0.7 mm bone gap in axolotl (white arrow). **g.** Skin stitching closed limb tissues and prevented loss of the bee wax strip.

Supplementary Figure 4. *Comparison of the bone healing process in mice and axolotl.*

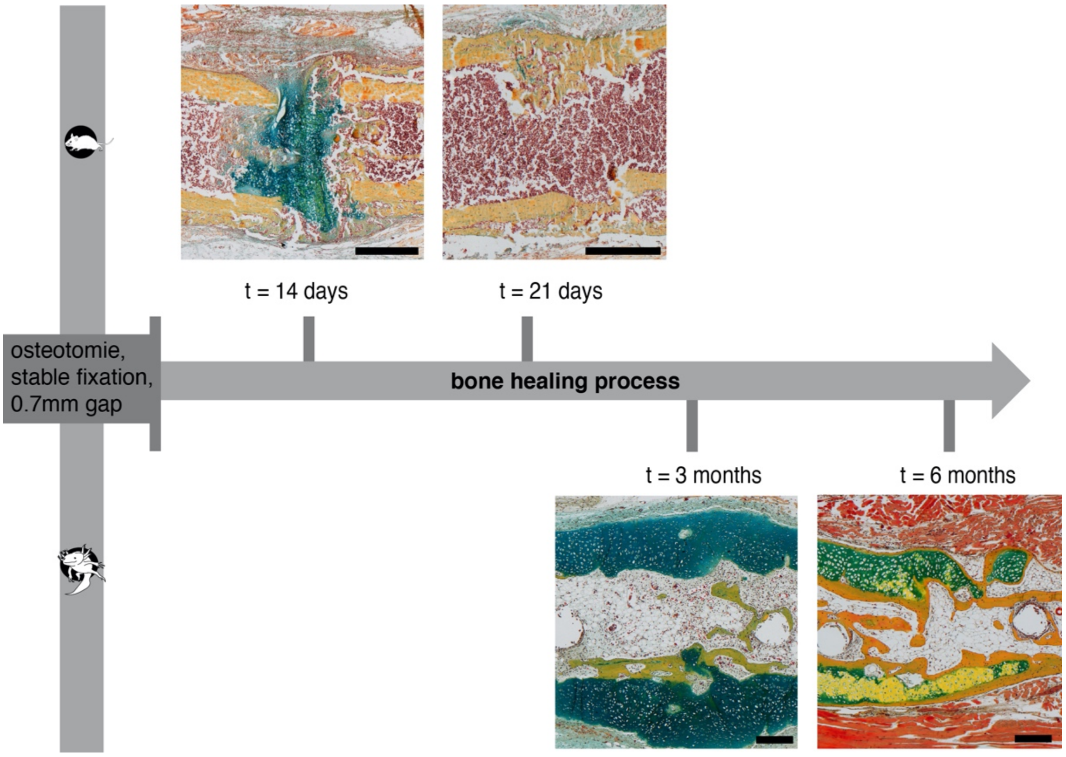

Both species underwent an osteotomy of the femur that received a stable fixation. The gap size was identical, 0.7 mm. An endochondral healing process was monitored – the healing underwent a chondrogenic soft callus phase. In mice a soft chondrogenic callus was visible 14 days after surgery (upper row left image: the green cartilage is filling the osteotomy gap, cortical bone is yellow and the bone marrow dark red with orange muscle surrounding the bone). A corresponding phase was observed in axolotl between 3 and 6 month (lower panel). In contrast to the mouse the cartilaginous callus surrounded the bone at the osteotomy side, not filling the gap. At 3 month the hyaline cartilage shows the green non mineralized matrix, at 6 months the chondrogenic callus becomes mineralized and the cartilage matrix turns yellow – the healing stage that precedes woven bone formation. In contrast at 21 days after surgery the mouse has bridged the osteotomy gap, cartilage has been replaces by bone (yellow) and even the bone marrow cavity is again reestablished – the healing is clearly in the remodeling phase (upper panel – right image). The healing in mice is clearly faster when considering the healing of a stable-fixated 0.7 mm gap. Movat‘s pentachrome staining, scale bars 500 µm.

Supplementary Figure 5. *SOX9 expression in femur fracture of 12 weeks old mice.*

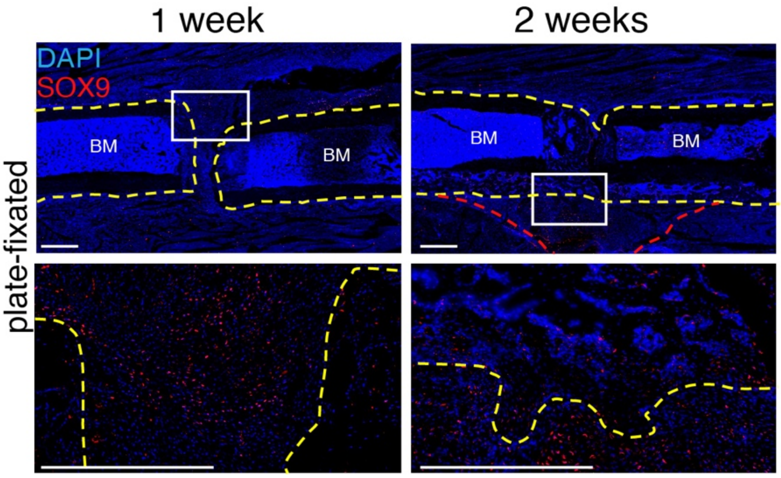

In murine fractures in young mice, SOX9-positive cells are present at 1 week post-fracture, and at 2 weeks bone bridging by woven bone is in progress. Scale bars 200 µm. Yellow dashed line - bone, red dashed line - callus, BM – bone marrow, M – muscle.

Supplementary Figure 6. *Comparison of SOX9 expression in axolotl fractured bones and amputated limbs to histological staining with Movat's pentachrome.*

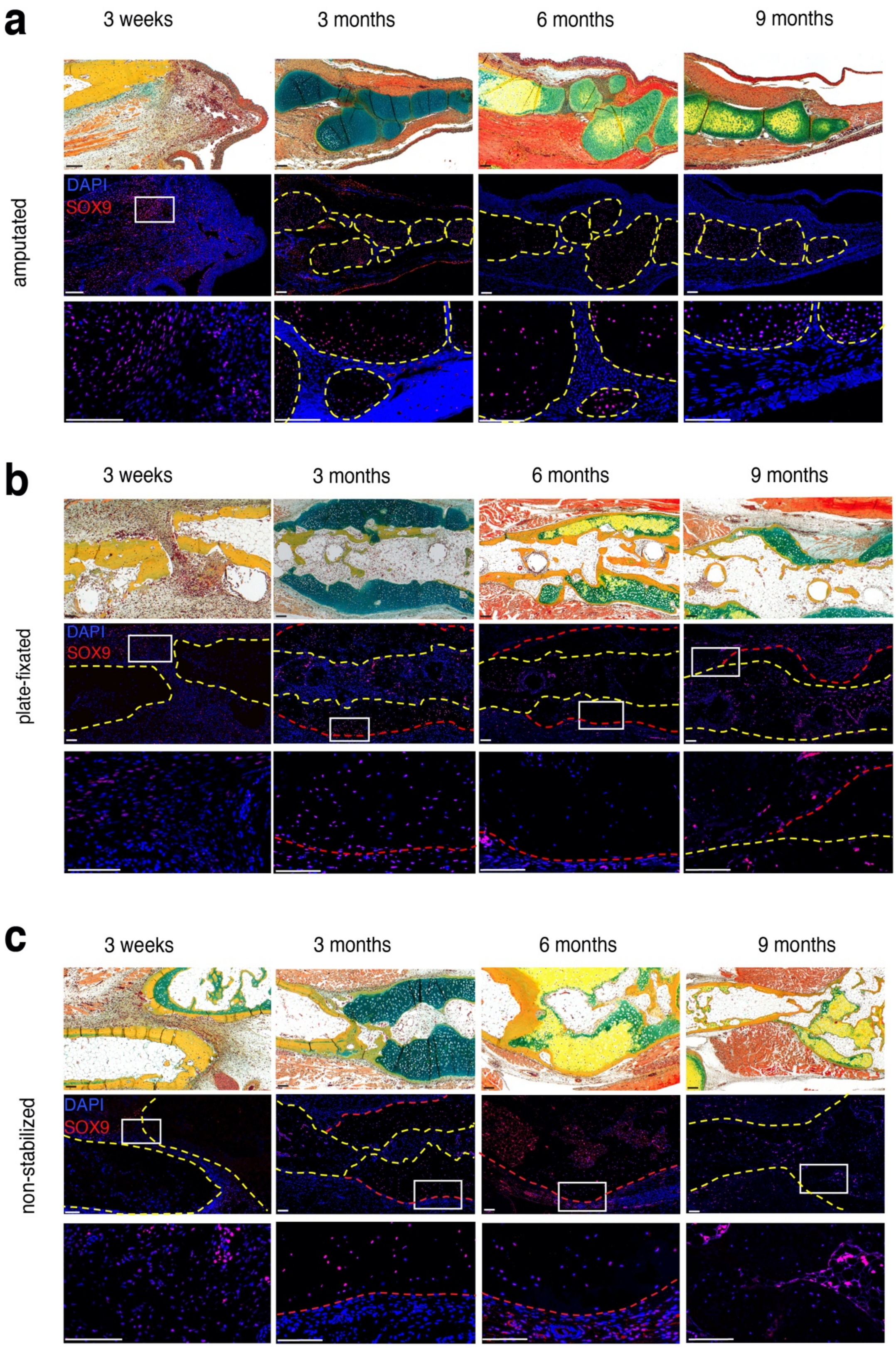

a. In axolotl amputated limbs, SOX9-positive cells are present in 3 weeks blastema and later in the cartilaginous condensations. **b, c.** In axolotl fixated and non-stabilized fractures, SOX9 expression is observed in pre-cartilage cells at 3 weeks post-fracture and in callus cells at 3 months post-fracture. SOX9 expression is retained even in the mineralized cartilage at 6 and 9 months post-fracture. Scale bars 200 µm. Yellow dashed line - bone, red dashed line – callus.

Supplementary Figure 7. *PCNA expression in axolotl amputated limbs and fractured bones.*

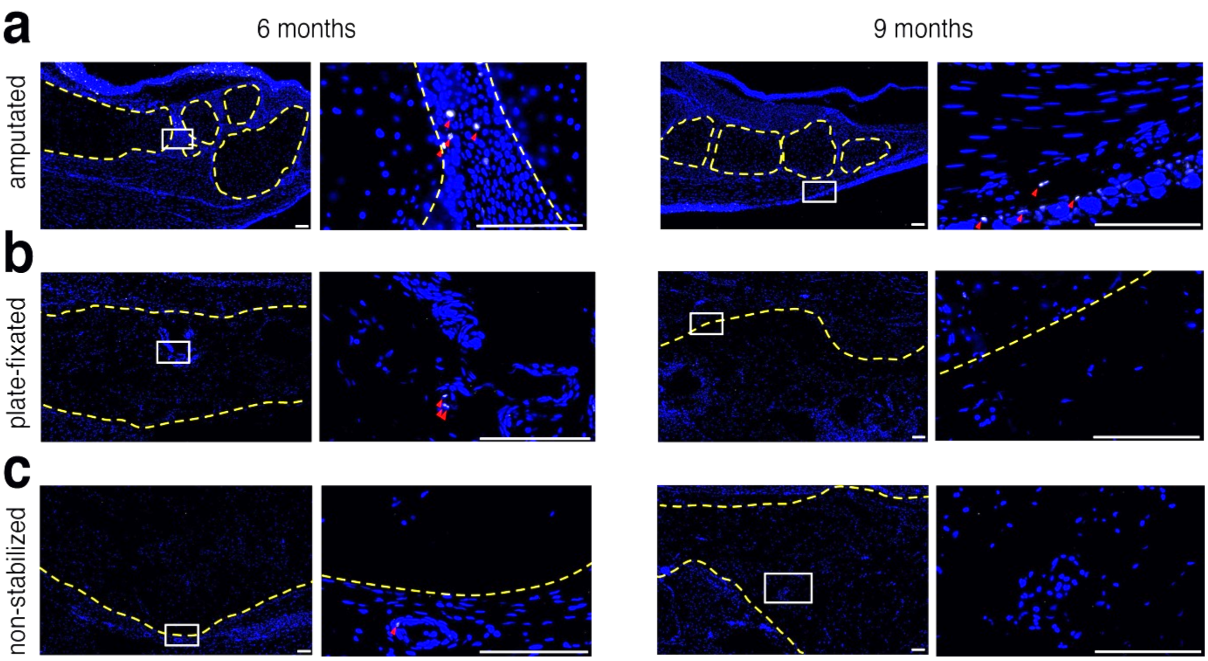

Representative samples of axolotl upper hind limbs stained with anti-PCNA (white) and DAPI (blue) 6 and 9 months post-surgery in axolotl amputated limbs (**a**), in plate-fixated fractures (**b**), and in fractures without fixator (**c**). Note very low number of PCNA^{+} cells. Scale bars 200 µm. Yellow dashed line - bone, red arrows point at PCNA^{+} cells.

Supplementary Figure 8. *PCNA expression in femur fracture of 12 weeks old mice.*

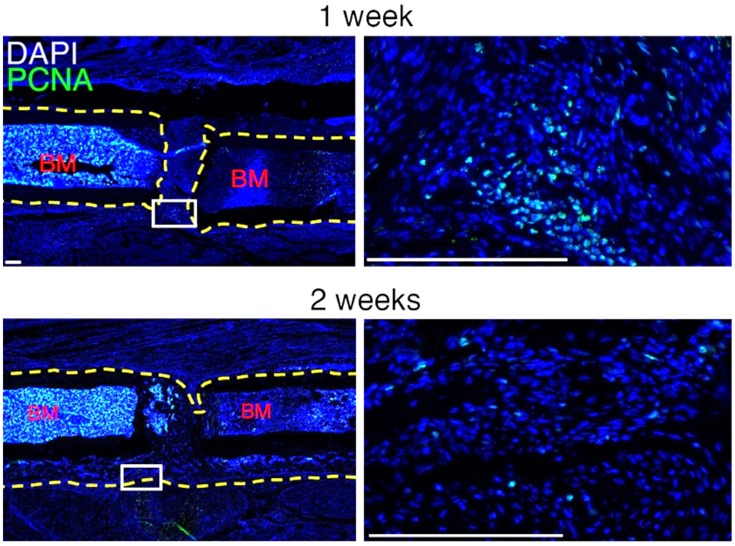

Fracture samples from young mice show many PCNA^{+} cells (green) in the proximity to injury site at 1 week post-fracture (similar to 1 year-old mice). At 2 weeks post-fracture, a few PCNA^{+} cells are retained in the newly formed bone. Scale bars 200 µm (left), 50 µm (right). Yellow dashed line - bone.

Supplementary Figure 9. *Newly formed callus in 6 months non-fixated axolotl fracture.*

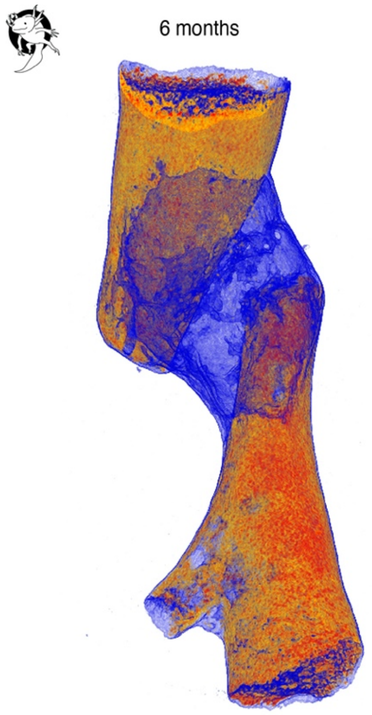

An non-fixated femur shown in a micro-CT image that distinguishes between the original bone (yellow-red) and the new formed callus (blue). The bone ends with the open bone marrow cavity are not aligned and a direct bridging is not possible. The newly formed callus connects the two bone fragments by forming a callus that attaches to the periosteum of the nearest bone surface of the opposite fragment. The newly formed blue callus bridges the area between the fragments that do not directly touch.

Supplementary Table. *Immunostaining conditions.*

Primary antibody	Supplier	Antigen retrieval		Antibody dilution	Blocking buffer
		Frozen (mouse)	Paraffin (axolotl)		
SOX9 ZRB5535 (Rabbit, anti-mouse, Zoomab)	Sigma-Aldrich	-	TE buffer, 40min, 80C	1:500, ON, +6C	5%BSA/PBS/ 0.1%Tween20
SOX9 AF3075 (Goat anti-human)	R&D Systems	not necessary	-	1:500, ON, +6C	5%BSA/PBS/ 0.1%Tween20
PCNA sc-56 AF647 (anti-rat)	Santa Cruz Biotechnology	Agilent Dako Target Retrieval Solution, Citrate pH 6.1 (S169984-2), 20 min, 80C	Agilent Dako Target Retrieval Solution, Citrate pH 6.1 (S169984-2), 40 min, 86C	1:500 for frozen slides, 2h, RT; 1:200 for paraffin slides, 2h, RT	5%BSA/PBS/ 0.1%Tween20
