# Supplementary material for: The specialist in regeneration—the Axolotl—a suitable model to study bone healing?
Source: NPJ Regen Med. 2022 Jun 30;7:35. doi: 10.1038/s41536-022-00229-4 (PMC9246919; doi:10.1038/s41536-022-00229-4)
Supplement: Supplementary file 2 — REPORTING SUMMARY [file 41536_2022_229_MOESM2_ESM.pdf]

nature portfolio

Corresponding author(s): Katharina Schmidt-Bleek

Last updated by author(s): May 15, 2022

# Reporting Summary

Nature Portfolio wishes to improve the reproducibility of the work that we publish. This form provides structure for consistency and transparency in reporting. For further information on Nature Portfolio policies, see our Editorial Policies and the Editorial Policy Checklist.

## Statistics

For all statistical analyses, confirm that the following items are present in the figure legend, table legend, main text, or Methods section.

| n/a | Confirmed | |
|---|---|---|
| ☐ | ☒ | The exact sample size ($n$) for each experimental group/condition, given as a discrete number and unit of measurement |
| ☐ | ☒ | A statement on whether measurements were taken from distinct samples or whether the same sample was measured repeatedly |
| ☐ | ☒ | The statistical test(s) used AND whether they are one- or two-sided<br>*Only common tests should be described solely by name; describe more complex techniques in the Methods section.* |
| ☐ | ☒ | A description of all covariates tested |
| ☒ | ☐ | A description of any assumptions or corrections, such as tests of normality and adjustment for multiple comparisons |
| ☒ | ☐ | A full description of the statistical parameters including central tendency (e.g. means) or other basic estimates (e.g. regression coefficient) AND variation (e.g. standard deviation) or associated estimates of uncertainty (e.g. confidence intervals) |
| ☐ | ☒ | For null hypothesis testing, the test statistic (e.g. $F$, $t$, $r$) with confidence intervals, effect sizes, degrees of freedom and $P$ value noted<br>*Give P values as exact values whenever suitable.* |
| ☒ | ☐ | For Bayesian analysis, information on the choice of priors and Markov chain Monte Carlo settings |
| ☒ | ☐ | For hierarchical and complex designs, identification of the appropriate level for tests and full reporting of outcomes |
| ☒ | ☐ | Estimates of effect sizes (e.g. Cohen's $d$, Pearson's $r$), indicating how they were calculated |

*Our web collection on statistics for biologists contains articles on many of the points above.*

## Software and code

Policy information about availability of computer code

| | |
|---|---|
| Data collection | n.a. |
| Data analysis | n.a. |

For manuscripts utilizing custom algorithms or software that are central to the research but not yet described in published literature, software must be made available to editors and reviewers. We strongly encourage code deposition in a community repository (e.g. GitHub). See the Nature Portfolio guidelines for submitting code & software for further information.

## Data

Policy information about availability of data

All manuscripts must include a data availability statement. This statement should provide the following information, where applicable:

- Accession codes, unique identifiers, or web links for publicly available datasets
- A description of any restrictions on data availability
- For clinical datasets or third party data, please ensure that the statement adheres to our policy

The data that support the findings of this study are available from the corresponding author upon reasonable request.



March 2021

# Field-specific reporting

Please select the one below that is the best fit for your research. If you are not sure, read the appropriate sections before making your selection.

☒ Life sciences ☐ Behavioural & social sciences ☐ Ecological, evolutionary & environmental sciences

For a reference copy of the document with all sections, see nature.com/documents/nr-reporting-summary-flat.pdf

# Life sciences study design

All studies must disclose on these points even when the disclosure is negative.

| | |
|---|---|
| Sample size | For the animal studies at least 3 and up to 7 animals per group and time point/ analyses were used - the sample sizes are detailed in the manuscript. The study is mostly descriptive in nature, therefore sample size calculation was not performed. Only the analyses for the size of fatty vacuoles of adipocytes has been statistically evaluated here n=100. |
| Data exclusions | Due to the descriptive nature of the data we did not exclude any data points. |
| Replication | Replication was done only with respect to the repetition of the surgery within the respective groups: amputation, fixation and non-fixed osteotomies. |
| Randomization | Animals were randomly allocated to the different groups. |
| Blinding | Blinding was not possible because the data clearly shows the treatment that has been applied. |

# Reporting for specific materials, systems and methods

We require information from authors about some types of materials, experimental systems and methods used in many studies. Here, indicate whether each material, system or method listed is relevant to your study. If you are not sure if a list item applies to your research, read the appropriate section before selecting a response.

## Materials & experimental systems

| n/a | Involved in the study | |
|---|---|---|
| ☐ | ☒ | Antibodies |
| ☒ | ☐ | Eukaryotic cell lines |
| ☒ | ☐ | Palaeontology and archaeology |
| ☐ | ☒ | Animals and other organisms |
| ☒ | ☐ | Human research participants |
| ☒ | ☐ | Clinical data |
| ☒ | ☐ | Dual use research of concern |

## Methods

| n/a | Involved in the study | |
|---|---|---|
| ☒ | ☐ | ChIP-seq |
| ☒ | ☐ | Flow cytometry |
| ☒ | ☐ | MRI-based neuroimaging |

## Antibodies

| | |
|---|---|
| Antibodies used | SOX9 ZRB5535 (Rabbit, anti-mouse, Zoomab) Sigma-Aldrich; SOX9 AF3075 (Goat anti-human) R&D Systems; PCNA sc-56 AF647 (anti-rat) Santa Cruz Biotechnology |
| Validation | SOX9 ZRB5535, SOX9 AF3075, PCNA sc-56- validated in mouse embryonic cryosections |

## Animals and other organisms

Policy information about studies involving animals; ARRIVE guidelines recommended for reporting animal research

| | |
|---|---|
| Laboratory animals | Ambystoma mexicanum (axolotl), DD strain; Mus musculus (mouse), C57Bl6J strain |
| Wild animals | n.a. |
| Field-collected samples | n.a. |
| Ethics oversight | axolotl: Magistrate of Vienna (GZ: 51072/2019/16, GZ 65248-2021-26); mouse: Magistrate of Vienna (GZ: 144706/2018/18). |

Note that full information on the approval of the study protocol must also be provided in the manuscript.
